# Flux Growth of Phosphide and Arsenide Crystals

**DOI:** 10.3389/fchem.2020.00186

**Published:** 2020-04-02

**Authors:** Jian Wang, Philip Yox, Kirill Kovnir

**Affiliations:** ^1^Department of Chemistry, Wichita State University, Wichita, KS, United States; ^2^Department of Chemistry, Iowa State University, Ames, IA, United States; ^3^Ames Laboratory, U.S. Department of Energy, Ames, IA, United States

**Keywords:** flux, crystal growth, phosphide, arsenide, salt flux, metal flux, self-flux

## Abstract

Flux crystal growth has been widely applied to explore new phases and grow crystals of emerging materials. To accommodate the needs of high-quality single crystals, the flux crystal growth should be reliable, controllable, and predictable. The selections of suitable flux and growth conditions remain empirical due to the lack of systematic investigation especially for reactions, which involve highly volatile components, such as P and As. Considering the flux elements, often the system in question is a quaternary or a higher multinary system, which drastically increases complexity. In this manuscript, on the examples of flux growth of phosphides and arsenides, guidelines of flux selections, existing challenges, and future directions are discussed. We expect that the field will be further developed by applying *in situ* techniques and computational modeling of the nucleation and growth kinetics. Additionally, leveraging variables other than temperature, such as applied pressure, will make flux growth a more powerful tool in the future.

## Introduction

The fundamental research goals of solid state chemistry, materials science, and condensed matter physics are to establish correlations between crystal structure and physical properties (Pamplin, 1980; Kanatzidis et al., 2005; The National Academies of Sciences Engineering Medicine, 2009). The single crystalline solids provide a suitable and simple platform due to the absence of grain boundaries and suppression and/or control of defects. Large, mm-sized or even bigger single crystals are indispensable for the characterization of anisotropic physical properties, such as magnetic, heat and charge transport, or optical properties (Phelan et al., 2012; Babu et al., 2018; Liu et al., 2018; Canfield, 2020). The scientific community has raised high standards for single crystal growth. Growth of single crystals should be fast, controllable, and capable of handling complex systems (Pamplin, 1980; Kanatzidis et al., 2005; The National Academies of Sciences Engineering Medicine, 2009; Canfield, 2020). One of the options is to grow crystals from a high-temperature molten media, flux, which is widely used for growth of different intermetallic, semiconducting, and insulating compounds of diverse chemical nature ranging from oxides and halides to metal alloys (Pamplin, 1980; Canfield and Fisk, 1991; Kanatzidis et al., 2005; Bugaris and zur Loye, 2012; Phelan et al., 2012; Juillerat et al., 2019; Canfield, 2020). The art of flux crystal growth is a combination of science and technology. The science part requires comprehensive knowledge of chemical bonding and reactivity of components and flux, combined with knowledge of the thermodynamics and kinetics of the growth processes. The technology aspect of the crystal growth is in its “trial and error” nature, which requires multiple attempts sometimes guided by the grower's intuition. *In situ* studies of the flux growth have provided insights regarding the structure of the liquid phase and cascades of solid–solid transformation occurring during heating and cooling (Shoemaker et al., 2014; Woo et al., 2018). Several general aspects of flux selection, together with examples and challenges, are discussed in this paper with focus on ternary and multinary phosphides and arsenides. The chemistry of phosphides and arsenides at elevated temperature is complicated because of the high vapor pressure of P or As. The volatility of P and As has resulted in poor exploration of many metal–phosphorus (arsenic) phase diagrams in the areas of high pnictogen content. Nevertheless, compounds with substantial P(As) content often exhibit interesting chemistry and useful practical properties, with potential applications as heat conductors, thermoelectrics, catalysts, and non-linear optical materials (Soheilnia et al., 2007; Lindsay et al., 2013; Dolyniuk et al., 2017; Nuss et al., 2017; Pöhls et al., 2017; Li et al., 2018; Owens-Baird et al., 2018, 2019; Woo et al., 2018; Coleman et al., 2019; Mark et al., 2019; Yu et al., 2019).

### Flux: General Considerations

**Figure 1:**
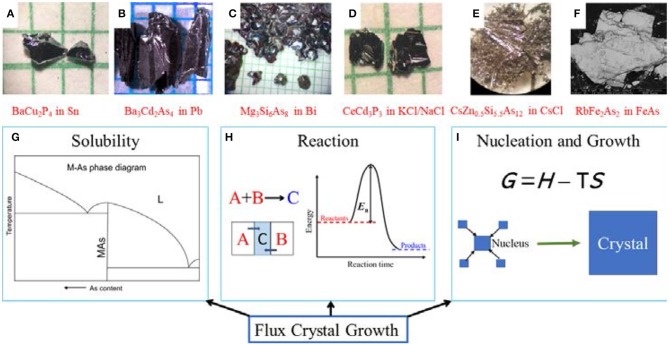
Top: Examples of phosphide and arsenide crystals grown in different fluxes (Wang et al., 2013; Dolyniuk et al., 2015; Woo et al., 2018, 2019a). **(D,F)** show unpublished crystals synthesized in Kovnir group. Bottom: main aspects of flux crystal growth. **(A)** BaCu_2_P_4_ in Sn, **(B)** Ba_3_Cd_2_As_4_ in Pb, **(C)** Mg_3_Si_6_As_8_ in Bi, **(D)** CeCd_3_P_3_ inKCl/NaCl, **(E)** CsZn_0.5_Si_5.5_As_12_ in CsCl, **(F)** RbFe_2_As_2_ in FeAs, **(G)** M-As phase diagram, **(H)** Solid-state reaction, **(I)** Nucleation and growth.

Various options exist for flux growth of inorganic crystals, such as metal flux, salt flux, or self-flux. Basic considerations for flux choice involve three aspects: solubility of reactants in flux, reaction path, and nucleation and growth of the target crystals (Figure 1, bottom). The presumptions about solubility of reactants in the flux are mainly drawn from the binary phase diagrams (Figure 1G). The flux crystal growth process is usually accompanied by chemical reaction, distinguishing itself from the growth of molecular compounds where recrystallization techniques prevail. In most cases, the flux plays a dual role in the crystal growth process: facilitating chemical reactions and aiding crystal growth (Figures 1H,I). In addition to the chemical and physical properties of the reactants and the products, interactions between the reactants and the flux should also be considered. The controllable nucleation and growth for a new system are challenging to achieve, due to the lack of systematic investigations. To compensate, a series of experiments are performed screening several flux/reagent ratios and heating/cooling rates. Owing to the limited size of this minireview, the comprehensive discussions of thermodynamics and kinetics are not covered here.

For exploring a new system, selecting a suitable flux is crucial. The following considerations of the flux properties are important:

#### Solubility

Moderate solubility of the target phase in the flux and a low-temperature dependence of the solubility are required. Low solubility of the reactants and the target phase will prevent chemical reactions and crystal growth. However, very high solubility will result in unsaturated solutions inhibiting the crystallization of the target phase. The temperature dependence of a target phase's solubility plays a critical role in the crystal growth process because it determines the cooling rate. For a new system or a new compound, solubility and its temperature dependence are often unknown and not easy to determine, but a few hints may be obtained by running several model experiments. Cooling rates as low as 0.4 K/h have been used, requiring weeks to complete the growth.

#### Inertness

The inertness of flux is required to avoid formation of stable phases between the flux and the reactants. Forming flux-containing compounds will shift the stoichiometry and disturb the nucleation process. Another aspect of flux inertness is undesirable doping of the target phase with flux components.

#### Melting Temperature

Large temperature ranges are ideal for crystal growth. Favorable fluxes have low melting temperatures coupled with high boiling points.

#### Toxicity

Non-toxic flux with low vapor pressure is safer and easier to handle for the environment and researchers. For example, Hg is rarely used as flux.

#### Container

To avoid incorporation of the container elements into the system and/or the target crystals, chemical reactions between flux and container are highly undesirable. If the flux has low solubility of volatile reactants (P, As) or the flux itself has a high vapor pressure, the container volume is another parameter that must be considered.

#### Removal

Flux should be removed after the growth is complete. Several methods including mechanical separation and polishing of crystals, high-temperature centrifugation of liquid flux, and dissolving flux at or near room temperature are frequently used. In the latter case the flux nature defines whether simple solvents (water, ethanol) or acid (HCl, H_2_O_2_/acetic acid) should be used. Dissolution requires the target phase to be stable in that media, limiting the choice of fluxes. Thus, KCl/NaCl is preferred over Sn flux for acid-sensitive materials due to the solubility of chlorides in water. Bi and Pb fluxes are not soluble in non-oxidizing acids and require the presence of hydrogen peroxide in the dissolving media.

#### Price

Flux is used in high excess in comparison to amounts of target materials or reactants. Thus, very expensive fluxes are always not the first choice especially with “trial and error” approaches. For example, Au/Si eutectic (T_melting_ = 636 K) might be a good flux, but it is rarely used due to the high price of gold.

#### Viscosity, Density, and Polarity

The high viscosity of flux may result in non-stationary mass transport, which brings no benefits to crystal growth. The densities of reactants and the flux should be comparable. Analogous to mixing water and benzene in a beaker, a phase separation may occur during flux crystal growth. In water/benzene combination not only density but also polarity of the liquids plays an important role. For flux synthesis, the rule of thumb is that metallic phases grow better from metallic fluxes (Sn, Pb, Bi), while semiconducting crystals are easier to grow from semiconducting fluxes (salts). This is not a strict rule, and exceptions are known.

The bottom line is that no single flux can satisfy all the criteria. However, these guiding principles can help to make rational flux choice for synthesis.

### Metal Flux Examples

Phosphorus and arsenic can form binary pnictides with almost all metals (excluding Hg and Bi) (Shatruk, 2019). Mercury has high vapor pressure and is toxic, thus bismuth should be the first choice for flux to grow phosphides and arsenides based on the inertness of flux criteria. Bi works well for arsenides due to the relatively high solubility of As in Bi. However, the solubility of P in Bi is quite low, and Sn flux is preferred for phosphides. Despite the existence of a number of binary Sn–P phases, multiple complex phosphides have been grown from Sn flux (Kanatzidis et al., 2005). Tin phosphides can be washed away with acid and have moderate melting points below 850 K. Binary tin arsenides have similar melting points to tin phosphides of ~870 K; however, Sn has somewhat limited success as a flux to grow arsenides; see example of *R*Co_2_*Pn*_2_ phases below. A common side-product of arsenide growth attempts in tin flux is SnAs binary with NaCl structure, which is different from SnP. The latter is a metastable phase with a unique crystal structure (Gullman, 1990). More systematic studies are required to figure out whether SnAs is an important intermediate, which affects crystallization processes or just a common side-product.

Pb was recently reported to form binary PbP_7_ (Schäfer et al., 2014), which demonstrates higher affinity of Pb to P compared to Bi, despite the exact phase diagram for the Pb–P system was not reported. Svilen Bobev et al. reported multiple successful crystal growths of both phosphides and arsenides from lead flux (Bobev et al., 2009; Saparov and Bobev, 2010; He et al., 2016). Finally, other metallic fluxes, such as Zn, Al, Ga, and In have been used for selected cases of phosphide and arsenide crystal growth.

#### BaCu_2_P_4_ in Sn Flux

Synthesis of twisted clathrate BaCu_2_P_4_ (Dolyniuk et al., 2015) is hampered by the existence of another clathrate with close composition, BaCu_2_P_3.75_. Thermal analysis confirmed that BaCu_2_P_4_ converts into BaCu_2_P_3.75_ upon heating prior to melting, which explains the experimental observations that high-temperature syntheses of BaCu_2_P_4_ always resulted in the formation of BaCu_2_P_3.75_. In turn, Ba and Cu starting materials are not active at low temperatures (<1,000 K) due to their high melting points, 1,003 and 1,353 K, respectively. To resolve this issue, flux growth was applied. P has high solubility in Sn (Zavrazhnov et al., 2018), which can be removed by centrifugation. The mm-sized BaCu_2_P_4_ crystals were successfully grown by Sn flux at 1,073 K (Figure 1A).

#### Mg_3_Si_6_As_8_ in Bi Flux

The Mg–Si–As ternary system has been overlooked for many years with only one theoretically predicted compound MgSiAs_2_ (Woo et al., 2018). Our synthetic efforts discovered three new ternary compounds, MgSiAs_2_, Mg_3_Si_6_As_8_, and Mg_3_Si_3_As_8_ (Woo et al., 2018; Wang et al., 2019). MgSiAs_2_ was confirmed to have chalcopyrite structure type, while the other two compounds crystallize in new structure types. The crystal growth of the Mg–Si–As system is challenging due to the combination of high vapor pressure of Mg and As and the inertness of Si at reaction temperatures. As discussed above, Bi is preferred over Sn flux for arsenides. Indeed, mm-sized red crystals of Mg_3_Si_6_As_8_ were obtained in Bi flux (Figure 1C).

#### Ba_3_Cd_2_As_4_ in Pb Flux

When exploring the synthesis of Ba_3_Cd_2_As_4_ (Wang et al., 2013), Cd and Pb flux were applied to grow crystals. Cd has a moderate range of operation between melting and boiling points, 594 and 1,038 K, respectively. Using Cd as a self-flux, the chances of incorporation of foreign elements were minimized. A disadvantage of Cd flux was the formation of binary admixture Cd_3_As_2_. Small crystals of Ba_3_Cd_2_As_4_ were grown from Cd flux with low yield. Larger crystals of Ba_3_Cd_2_As_4_ were grown from Pb flux. Pb has a comparable melting point and much higher boiling point than Cd, 603 and 2,023 K, respectively. The Ba, Cd, and As reactants have reasonable solubility in Pb at elevated temperatures. Mm-sized crystals of Ba_3_Cd_2_As_4_ were grown in Pb flux (Figure 1B).

#### RCo_2_Pn_2_ (R = Rare-Earth Metal; Pn = P and As) in Sn and Bi Fluxes

In the three aforementioned examples, direct reactions of elements produced the polycrystalline samples of the target phases. Often this is not the case, and flux is used to overcome high reaction barriers. For example, reactions of neat La, Co, and P resulted in a mixture of stable binary phosphides, LaP, and CoP/Co_2_P, which would not react with each other preventing formation of La–Co–P ternaries. Sn flux resolves these issues resulting in mm-sized crystals of *R*Co_2_P_2_, which allows for establishing of the structure–magnetic property relationships (Kovnir et al., 2010, 2011a,b; Kovnir et al., 2013; Thompson et al., 2014a; Tan et al., 2017). When switching to isostructural arsenides, tin flux failed to produce any *R*Co_2_As_2_ phases. Instead Bi flux was successfully applied (Thompson et al., 2011, 2014b; Tan et al., 2016a, 2018). Detailed structural characterizations revealed that Bi is capable of partially replacing *R*^3+^ cations, probably due to its trivalent nature (Thompson et al., 2011). No evidences of Sn incorporation into crystals of *R*Co_2_P_2_ were found. However, divalent Sn was reported to partially replace Ba^2+^ in isostructural *A*Fe_2_As_2_ superconductors (*A* = Ba, K) (Mathieu and Latturner, 2009).

#### GeP in Sn and Bi Fluxes

Incorporation of the metal flux components into the crystal structure is one of the reasons for discarding certain fluxes. Elemental Ge and P will not react with each other at the temperatures at which binary GeP is stable, thus calling for flux application. The growth of GeP van-der-Waals semiconductor crystals were achieved from Sn flux (Lee et al., 2015). The detailed structural characterizations show that a significant degree of disorder in both Ge and P sites is introduced by partial replacement of Ge with larger Sn atoms. Sn doping resulted in drastic decrease in melting/decomposition temperature by 228 K compared to the pristine GeP. Similarly, over an order of magnitude increase in electrical resistivity was detected for Sn-doped GeP. Undoped GeP was successfully synthesized using Bi flux instead of Sn. No incorporation of Bi was detected by SEM/EDS and crystallographic investigations (Lee et al., 2015).

### Salt Flux Crystal Growth Examples

Many inorganic pnictide crystals can be grown in salt fluxes. Salt fluxes are high-temperature ionic liquids (Table 1). The salt flux can be inert (NaCl/KCl) or reactive (CsCl, AuCl, ZnCl_2_). A huge advantage of salt flux is the simplicity of removal by solvent wash, especially by water. Owing to the lack of knowledge about elemental solubility in salts, the selecting of salt flux is an empirical process, until appropriate phase diagrams are made. An example of crystals of CeCd_3_P_3_ grown in NaCl/KCl inert salt flux is shown in Figure 1D. Reactive fluxes often can be applied to overcome inertness of a transition metal. Thus, Ba*T*_2_P_4_ and Ba*T*_2_P_3.75_ (*T* = Cu, Ni, Au) can be produced starting from *T*Cl or *T*Cl_2_ precursors and excess of Ba (Kovnir et al., 2011; Fulmer et al., 2013a,b; Dolyniuk et al., 2015). Metal chlorides serve as source of *T* element due to Ba + 2*T*Cl → BaCl_2_ + 2*T* reactions. Simultaneously, the mixture of barium and transition metal chlorides serves as a flux media.

**Table 1:** Selected phosphide and arsenide crystals grown from flux.

**Flux type**	**Flux role**	**Flux**	**Compounds**	**References**
Metal	Inert	Bi	*A*_2_Co_12_As_7_ (*A* = Ca, Y, Ce–Yb)	Tan et al., 2016b
	Inert	Pb	Ba_7_Ga_4_*Pn*_9_ (*Pn* = P, As)	He et al., 2016
	Dopant	Sn	Sn*_*x*_*B_1−x_P	Woo et al., 2016
	Inert	Ga	*R*_4_Mn_2_As_5_ (*R* = La–Pr)	Tabassum et al., 2015
	Source of In	In	Eu_3_In_2_P_4_	Jiang et al., 2005
Salt	Inert	NaCl/KCl	LaCu_4_P_3_	Wang et al., 2015
	Source of Au	AuCl	BaAu_2_P_4_	Fulmer et al., 2013a
	Inert	NaCl/KCl	BaCuZn_3_As_3_	Ozawa and Kauzlarich, 2003
	Source of Mg	MgI_2_	La_2_Mg_3_SiP_6_	Wang et al., 2017
	Source of Zn	ZnCl_2_	La_7_Zn_2_P_11_	Wang et al., 2018
Self-flux	Self	KAs	K_2_Cr_3_As_3_	Bao et al., 2015
	Self	FeAs	CaKFe_4_As_4_	Meier et al., 2016
	Self	CoAs	CaCo_2_As_2_	Cheng et al., 2012

Finally, salt flux may be used to reduce the reactivity of a reactant. Metallic Cs is extremely reactive, challenging to handle, and requires a glovebox due to its pyrophoric nature. Instead, the CsCl + Mg combination can be safely handled in air. CsCl is used as a flux and a source of Cs while producing a mixture of Mg and Cs chlorides that are removed by water washing after synthesis (Figure 1E) (Woo et al., 2019a).

#### La_2_Mg_3_SiP_6_ in MgI_2_ Flux

As summarized in Table 1, salt flux was useful to grow compounds in La–*M*–P systems (*M* = Zn, Cd, Cu, Mg, Si). The crystal growth of La_2_Mg_3_SiP_6_ was impeded by the combination of inertness of La and Si and high reactivity and vapor pressures of Mg and P (Wang et al., 2017). Attempts to grow La_2_Mg_3_SiP_6_ using Sn or Zn metal flux were unsuccessful. Changing to KCl/NaCl flux resulted only in a mixture of Zn_3_P_2_ and SiP_2_. Finally, MgI_2_ was proven to be proper flux for crystal growth of La_2_Mg_3_SiP_6_. Partial decomposition of MgI_2_ at reaction temperatures and release of iodine, which may serve as a transport agent might play a role in promoting reactivity of binary phosphides.

### Self-Flux

The crystal growth by self-flux (flux composed solely of elements present in the target crystals) has the lowest chances of incorporating foreign elements. The self-flux is usually pre-synthesized, examples are provided in Table 1. When exploring the synthesis of a ternary system ABC, all possible binary compounds, AB, AC, BC can be considered as self-flux if they meet the following criteria: facile synthesis, low melting and high boiling points, easy removal. For example, Ca*A*Fe_4_As_4_ (*A* = K, Rb, Cs) superconductors were initially discovered as polycrystalline samples, which only exist in a narrow temperature window (Iyo et al., 2016). Afterward, large crystals were grown from FeAs self-flux (Meier et al., 2016). A special crucible design, such as the Canfield crucibles, is very useful for separation of large crystals from flux (Canfield, 2020).

### Transport Growth in Flux

Transport reactions in a liquid flux have a characteristic temperature gradient. At the hot end, reactants dissolve or form mobile species that migrate toward the cold end and crystallize in the target phase. This stands for endothermic equilibrium reaction, while for exothermic reaction, the transport happens from cold to hot end. Provided that the temperature gradient is consistent and includes the crystallization temperature of the target phase, crystal growth occurs on the cold end while simultaneously consuming reactants on the hot end, similar to the vapor transport reactions. Transport reactions do not require full solubility of reactants in the flux, allowing for a wider choice of fluxes and larger mass loadings of the starting materials, resulting in larger quantities of produced crystals. Transport reactions allow certain flexibility when selecting a flux because the solubility of reactants may not be as crucial. In turn, the viscosity and associated mass transport apply stricter constraints to the flux selection. This method is relatively new but has been demonstrated for crystal growth of antimonides, halides, chalcogenides, and transition metal chalcophosphates with MP*Q*_3_ (*Q* = S, Se) compositions (Yan et al., 2017). The development of transport reactions for crystal growth of phosphides and arsenides is currently underway. For liquid transport, presumably all of the fluxes previously discussed could allow crystal growth, which indicates that temperature gradients can be a useful variable when applied correctly. In a more in-depth review, Yan et al. (2017) demonstrated several examples of self-flux and salt flux used for transport growth.

## Discussion and Perspective

As discussed above, a researcher has several flux-based methods to grow single crystals of desired phosphides and arsenides. There is no universal, one-size-fits-all, flux method and no guarantee that for any given phosphide or arsenide the flux growth of cm-sized crystals can be developed. More systematic studies of flux growth mechanisms are required to make the latter statement false. Since the reaction and crystal growth are performed within a sealed environment to contain vapor pressure of pnictogens and prevent oxidation by air, the observation of reaction and crystal growth processes are limited. Many assumptions based on simple binary phase diagrams have to be made, which are not always correct, and not all binary phase diagrams are fully developed. Computational modeling of liquid-to-solid transformations in selected areas of complex phase diagrams may help reduce the number of trials for crystal growth. Recent developments of *in situ* techniques, TEM, and elastic scattering at synchrotron and neutron sources, revealed a lot of hidden processes occurring during flux synthesis, thus allowing for more rational design of flux reactions and crystal growth (Shoemaker et al., 2014; Woo et al., 2018, 2019b; Vasquez et al., 2019). Finally, computationally cheap methods, such as machine learning (Yao et al., 2019), can be applied to build bridges between “trial and error” and fully rational approaches. Results of unsuccessful trials, which are documented only in lab notebooks are required to train machine learning models and reveal hidden relationships.

Another challenge to be addressed is an adaptive control of crystal growth. Currently, most flux crystal growth relies upon spontaneous crystallization processes by manipulating temperature, initial reactants/flux ratios, and cooling rates. A design of a new type of flux crystal growth furnace, which can balance the inert atmosphere requirement and controlled growth with adaptive feedback loop, may provide larger and better quality crystals. The last to be mentioned, flux crystal growth under extreme conditions such as high temperature–high pressure remains essentially unexplored. Sparked by the recent discovery of almost room temperature superconductivity in LaH_10_ superhydride, there are rich opportunities to explore new phases and reaction mechanisms under applied pressure (Geballe et al., 2018). However, the challenges due to the presence of extreme conditions and necessity to work in limited sample environments are substantial. So far, crystals of superhydrides have not been produced and studied. Maybe high-temperature and high-pressure flux can help to grow crystals of superhydrides?

## Author Contributions

The manuscript was written through contributions of all authors. All authors have given approval to the final version of the manuscript.

### Conflict of Interest

The authors declare that the research was conducted in the absence of any commercial or financial relationships that could be construed as a potential conflict of interest.
